# Feasibility of a secondary school-based mental health intervention: *Reprezents’ On The Level*

**DOI:** 10.1186/s13034-022-00534-2

**Published:** 2022-12-09

**Authors:** Natalie Bisal, Jilly Gibson Miller, Christine Cox, Shane Carey, Liat Levita

**Affiliations:** 1 University of Sheffield, Sheffield, UK; 2 Reprezent Youth Development Organization, London, UK; 3 University of Sussex, Sussex, UK

**Keywords:** Adolescents, Secondary schools, Interactive digital mental health intervention, Cognitive behavioural framework, Feasibility

## Abstract

**Aims:**

There is a need for innovative school-based mental health interventions to promote good mental health, healthy coping strategies, and engagement with support services. Consequently, Reprezent, a youth development organization, with mental health professionals and young people co-developed an online mental health intervention show, On The Level (OTL). This study assessed the acceptability and feasibility of delivering OTL to young people (aged 11–18 years) in 36 secondary schools across London and Essex, UK.

**Methods:**

OTL was delivered online as part of the school curriculum, in classrooms at timepoint 1 (T1, 50 min). Follow-up data was collected at timepoint 2 (T2) 4–6 weeks later, during a 20-min OTL review show. For interactive OTL elements and data collection participants logged into an online survey. Measures of acceptability and engagement, mental health and well-being outcomes and intervention evaluation were taken at T1 and T2. We also assessed the feasibility of implementing the OTL intervention in secondary schools.

**Results:**

10,315 participants received the intervention (T1) and 3369 attended the follow-up session (T2), this high attrition, and potential selection bias, was due to only 30% of schools being able to take part in T2. Rates of acceptability were high among young people and school staff. At T1, 88% found OTL engaging, and 84% felt more confident they had the tools to help them better manage stress and anxiety. At T2, 66% viewed mental health in a more positive way, and 71% had better understanding of how to maintain good mental health. Rates of engagement with mental health tools and services were good, and significant reduction in levels of stress were found 4–6 weeks after the OTL show (T2). The low mental health and well-being indices reported by the school children at baseline strongly support the need and use for a mental health intervention such as OTL in secondary schools.

**Conclusion:**

These findings indicated good feasibility and acceptability of OTL intervention and support the delivery of the OTL mental health intervention at UK-based secondary schools to educate young people about mental health and well-being and give them the necessary tools to support their mental health.

**Supplementary Information:**

The online version contains supplementary material available at 10.1186/s13034-022-00534-2.

## Background

Adolescence is a prolonged developmental period and a key time for identity formation, developing resilience, self-awareness, and self-regulation skills [38, 45, 54]. During this time some adolescents show increased vulnerability to experiencing, academic and school-related problems [12, 30, 51], and social pressures [44], and increased vulnerability for poor mental health [2], with symptoms of lifelong mental illness typically developing prior to the of age of 25 [4, 14, 15, 31].

It has been suggested that the stress and anxiety associated with the various aspects of the COVID-19 pandemic would result in even higher levels of poor mental health of young people [26, 37]. Indeed, during the global COVID-19 pandemic, it has been increasingly recognised that young people worldwide have been adversely affected [8, 18, 25]. The pandemic, and especially the various lockdowns, have resulted in acute loss of normal social connections [8, 18], mental health support [55], and greater uncertainty about the future [57] for young people, with a number of studies reporting an increase in mental health issues such as anxiety, stress, and depression by some young people since the beginning of the pandemic [28, 33, 35, 55]. This has brought to the fore, the pressing need to address as a priority, the mental health and well-being of young people [22, 56].

Schools have an important role to play in the promotion of mental health and wellbeing, as a universal setting whereby positive mental health and wellbeing is promoted and early identification, support and intervention could be provided for those with specific mental health needs [7, 20]. Owing to the unique positioning throughout the formative years of childhood and adolescence, schools can play a central role in making mental health and wellbeing support more accessible, increasing mental health knowledge and reducing stigma associated with seeking mental health support [20], reported as key barriers to seeking and accessing mental health support reported among young people [32]. Providing effective mental health knowledge and support at school has challenges owing to a lack of staff mental health training, resources availability, and time constraints that exist within the school system [34, 52] and during the pandemic and lockdowns, issues with being able to deliver face-to-face mental health support to students in schools [10, 16, 42]. Furthermore, much of the research investigating school-based mental health interventions is reportedly based on small sample sizes [19, 21], a lack of adherence and engagement measures [3] and potential social desirability bias where interventions were delivered by class teachers [24].

There is therefore a need for innovative school-based interventions that aim to raise awareness of mental health among young people and to promote healthy coping strategies and engagement with support services. Mental health support, education, and tools are essential in addressing the potential impact of COVID-19 on young people’s mental health and well-being [29, 42, 50]. To address this Reprezent, a youth advocacy group, together with mental health professionals and young people co-developed an online mental health show, On The Level (OTL). Reprezent is a youth development organisation and media platform, helping young people realise their full personal potential through core social, emotional and communication skills. They run training and development programmes and champion youth culture through a London-wide radio station, REPREZENT 107.3FM. The OTL intervention components were based on a cognitive-behavioural framework comprising elements of psychoeducation, role modelling, monitoring stress/anxiety, practical support, safeguarding, and cognitive behavioural strategies. Reprezent delivered the OTL intervention online via an interactive show in schools across London and Essex, UK. Each event is presented by young people who have been trained by Reprezent to provide mental health education and support to young people.

## Current study

The overarching aim of this feasibility study was to investigate the feasibility of the On the Level (OTL) Mental Health intervention for a future larger RCT study that will examine the effectiveness of OTL delivered to young people in secondary schools. The first objective was to assess the acceptability, engagement and demand for the OTL intervention among young people and the school staff who facilitated the intervention in class. The second objective was to determine the feasibility of implementing the intervention in secondary schools. The third objective was to assess the feasibility of the study methods utilised. Finally, we assessed the feasibility of a range of potential outcome measures.

## Methods

### Design and procedure

**Fig. 1:**
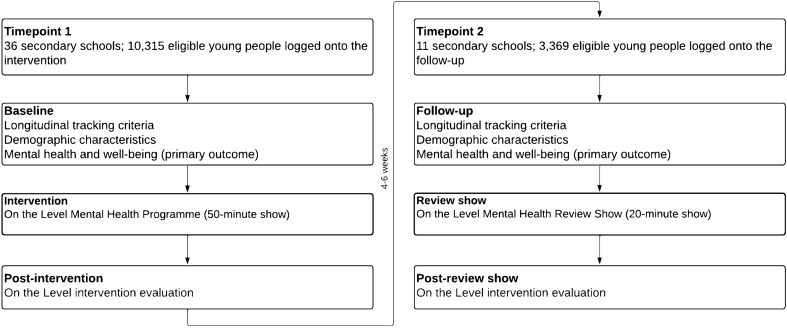
Survey measures at timepoints 1 and 2

A single-arm feasibility study design was adopted for this study. Data was collected between March and July 2021 at two timepoints; T1 and follow-up (T2) 4 to 6 weeks after T1 (see Fig. 1). The data collection period followed the third COVID-19 lockdown in England. Assessment of mental health and demographic information was collected at T1 (pre-intervention, just before the start of the OTL show and follow-up (T2, 4 to 6 weeks after T1). Participants’ views of the acceptability of the OTL intervention and self-reported engagement with mental health services and tools were assessed at post-intervention and follow-up. Assessments of acceptability and ease of use data was collected from the staff members who facilitated the OTL intervention during class at post-intervention. Staff members who facilitated the intervention during class provided feedback, via Survey Monkey (an online survey software, https://www.surveymonkey.co.uk/) on measures of acceptability, ease of use and demand for the OTL intervention in the school.

Setting-level recruitment took place between October 2020 and March 2021 from 36 secondary schools. Information about the project was sent to schools via email or a phone call from the study team. Consent to deliver the OTL intervention was obtained from headteachers, pastoral leads or year heads prior to intervention delivery. The 50-min intervention was delivered in classrooms during school hours, presented as an online show at timepoint 1 (T1). Follow-up data was collected at timepoint 2 (T2) during a 20-min OTL review show. Data was collected using an on line survey delivered via Slido (an online interactive polling and questions platform, https://www.slido.com), to which participants were required to log into on their mobile phones, tablet or other online devices available at the school. School staff members oversaw the delivery of the intervention through supporting students to access the intervention and complete the survey measures whilst ensuring the school safeguarding procedures were followed during and after the intervention. Staff members were supported with resources and instruction from the team at Reprezent. Table 1 provides an overview of the OTL mental health intervention and the review show content.

**Table 1:** On the Level Intervention Techniques and Content

Technique	Example
Psychoeducation	● Understanding one’s emotions, and recognizing when these are normative responses to stress/challenge, and when they are not ● Normative information about mental health in young people ● Cognitive behavioural symptoms of stress and anxiety (thoughts, emotions, feelings and behaviours) ● Critical voices and negative automatic thoughts ● Understanding one’s own development/body (Psychobiology, brain development being an on-going process throughout the adolescent years) and role of amygdala in producing and regulating levels of anxiety (e.g., fight or flight response)
Role Modelling	● Intervention presented and co-written by young people (members of Reprezent) ● Presenters (e.g., Stormzy, a celebrity presenter) and other young peers regularly offer their own stories and strategies for dealing with their anxiety and stress and give advice to audience (also Covid-19 related content of different experiences both good and bad of the pandemic and lockdowns) ● Demonstration of practical strategies by presenters
Monitoring stress/anxiety	● Identifying personal level of stress on the ‘Stress dial’ (red, orange, yellow, green) ● Recognising one’s personal responses to stress and anxiety (implode/explode)
Offering practical support/ Safeguarding	● Demonstration of age-appropriate online support resources (Woebot, Kooth) ● Action to take in emergency
Practical Cognitive behavioural strategies	● ‘Mental health 5-a-day’ (Awareness, breathing, relaxation, affirmation, movement) ● Compassion/kindness to others
Written information	● Custom made resource pack for schools

### Participants

As a universal intervention, all young people in participating classes were invited to take part in the study. Participants were young people aged between 11 and 18 years who attended one of 36 secondary schools in London or Essex, UK (Demographic information, Table 3).

### Intervention

The OTL intervention aimed to encourage young people to manage their mental health and wellbeing through raising awareness and promoting increased self-awareness and knowledge about their own emotions and development including young people were encouraged to engage with mental health conversations and introduced to tools for practical support. This was a collaborative project between University of Sheffield psychology researchers and Reprezent, a popular youth radio station designed for and presented by young people. The intervention was co-developed with young people, mental health professionals, teachers and presented by young people employed by Reprezent. The intervention was filmed in advance and then broadcast in classrooms, comprising a range of NHS commissioned digital health services (e.g., Kooth online counselling service), digital platforms (Slido) and interactive intervention elements to promote engagement and relevance among young people during the show. The main components, tools and techniques delivered during the intervention are shown in Table 1. Specifically, the intervention components were based on a cognitive-behavioural framework comprising elements of psychoeducation, role modelling, monitoring stress/anxiety, practical support, safeguarding, and cognitive behavioural strategies. Participants were encouraged to reflect on their own mental health experiences and methods of coping during the intervention by responding to various questions prompted throughout the intervention (presented in Additional file 1: Table S1). Support included preventive mental health tools (e.g., a ‘mental health 5-a-day’ tool co-developed with young people specifically for the OTL mental health intervention) and mental health services for young people experiencing mental health difficulties, such as Kooth and Woebot. The ease of use and guidance for the school staff members was a priority in light of the challenges and time constraints following the closure of schools across England during the pandemic.

### Study measures

Participant demographic information (age, gender, ethnicity, year group, school and longitudinal tracking data which included the first two letters of first name and month of birth) was collected at baseline and follow-up. The outcome measures were collected at the three timepoints (baseline, post-intervention and follow-up). A summary of the study measures, approaches and analyses for the study objectives is presented in Table 2.

**Table 2:** Overview of analyses for the feasibility study objectives

Objective	Outcomes	Measures	Analysis
1. Acceptability, engagement and demand for the OTL intervention	Acceptability	- Participant acceptability, satisfaction, intent to use services/tools, evaluation of OTL (T1 and T2 post-show), and preference for future OTL intervention - School staff member acceptability, ease of use, and importance and need for OTL intervention	- Descriptive statistics - Frequencies
	Engagement	- Participant use of support services and/or tools (T2)	- Descriptive statistics - Frequencies
2. Feasibility of implementing the intervention	Recruitment (setting-level)	- % schools contacted, interested, and participated	- Descriptive statistics - Frequencies
	Reach (individual-level)	- % potentially eligible, excluded, and participated	- Descriptive statistics -Frequencies
	Retention (setting- and individual-level)	- No. of enrolled schools returned to follow-up - No. of enrolled participants that completed follow-up	- Descriptive statistics - Frequencies
	Differential attrition	- Comparison of (a) responders, partial- and non-responders, and (b) matched and non-matched participants	- Descriptive statistics - Frequencies - Chi-Square
3. Feasibility of study methods	Data collection	- Proportion of participant responses (responders) and incomplete responses (partial and non-responders) for baseline, post-intervention and follow-up survey measures - Data collected as intended at key timepoints (pre-, during, post-show)	- Descriptive statistics - Frequencies
	Longitudinal tracking	-% of participants matched at baseline and follow-up	-Descriptive statistics - Frequencies
4. Need for mental health intervention & feasibility of outcome measures	- Mental Health outcome pre-intervention - Differences observed in mental health outcome measures (between T1 and T2)	- Anxiety^a^, Depression^a^, Life satisfaction^a^, Well-being^b^, Perceived stress^c^, Locus of control^d^, Sleep, Screen time	- Descriptive statistics - Median (IQR) - Wilcoxon Signed-Rank

*OTL* On the Level

^a^ 1-item measure

^b^ The World Health Organization Five Well-Being Index (WHO-5)

^c^ The Perceived Stress Scale (PSS-4)

^d^ Internal Locus of Control scale (LOC-3)

### Acceptability, engagement and demand for the OTL intervention (objective 1)

Acceptability and engagement with the OTL intervention assessed at post-intervention and follow-up (see Additional file 1: Table S2 for survey questions). To assess the acceptability of OTL at T1 participants completed an evaluation survey immediately after the intervention.

This included questions about how engaging participants found the intervention, their perceived confidence in having the tools to manage stress and anxiety, helpfulness and intent to use the mental health tools and/or services presented in the show, and their confidence in offering advice to a friend who was feeling stressed. Assessment of the acceptability of OTL at T2 included questions related to participant’s views of mental health and their understanding of what is needed to maintain good mental health since the intervention. Engagement was assessed at T2 via self-reported use of the mental health tools and services that were presented in the OTL intervention.

The school staff member survey included questions to assess the staff member’s views of intervention acceptability, ease of use and the importance and need for a mental health intervention at T1. The survey included questions related to data collection procedures, ease of use, and importance and recommendation of OTL to other schools (see Additional file 1: Table S3 for the survey questions).

### Feasibility of implementing the intervention (objective 2)

Recruitment was assessed at setting-level by the percentage of secondary schools who were contacted, raised initial interest, enrolled, and took part in the study. Reach was assessed at individual-level by the percentage of participants who were potentially eligible to take part, excluded, and participated in the study. Retention was assessed at setting-level by the percentage of schools who returned at follow-up and at individual-level by the percentage of participants that completed follow-up (T2).

Differential attrition was assessed by differences in demographic characteristics between participants who were (a) full responders, and partial and non-responders and (b) matched (T1 and T2) and non-matched (T1 only) participants, on mental health and well-being outcome measures collected at baseline and follow-up.

### Feasibility of study methods (objective 3)

Data collection methods were assessed by the proportion of participant responses (responders) and incomplete responses (partial or non-responders) for measures collected at baseline, post-intervention and follow-up. Completion rates for each survey varied as response to the survey items was voluntary. Data collection adherence was assessed by the extent to which data was collected as intended at the key timepoints (i.e., baseline, post-intervention and follow-up). The procedure of longitudinal tracking was assessed by matching participants on a range of criteria (age, gender, ethnic background, school year, school) taking into consideration possible changes in age and school year between T1 and T2.

### Assessing intervention need and feasibility of potential outcome measures (objective 4)

Mental health and well-being outcomes were collected just before the start of OTL intervention show at T1 (baseline) and before the start of the review show at T2 (follow-up). At baseline (T1) self-report of mental health indices were used to assess the need for a mental health intervention in secondary school pupils. Differences between mental health outcomes were compared between T1 and T2 to assess the feasibility of using these outcome measures in a future random control trial (RCT) study. The mental health and well-being survey comprised of 20 questions related to mental health, well-being and health-related behaviours.

*Mental health.* To assess anxiety presence, we used the 1-item measure: “Over the past week have you felt anxious?” on a 6-point Likert scale from 0 = ‘At no time’ to 5 = ‘All of the time’ [48].

To assess depression presence, we used the 1 item measure: “Over the past week have you felt depressed?” on a 6-point Likert scale from 0 = ‘At no time’ to 5 = ‘All of the time’ [48].

Perceived stress was measured using the 4 item Perceived Stress Scale [6]. The scale contains four items rated on a 5-point Likert scale from 0 = ‘never’ to 4 = ‘very often’. Total scores range from 0 and 16 a higher score indicates higher perceived stress. Stress was also measured using a stress dial item which asks participants to rate which level they felt they were on the stress dial from ‘Green – Healthy’, ‘Yellow – Coping’, ‘Orange – Struggling’, ‘Red – Critical’. This measure was co-developed as part of public involvement with secondary school students for the purposes of assessing different levels of stress, ranging from ‘healthy’ to ‘critical’, in a relevant and acceptable way.

*Well-being.* To assess life satisfaction we used the 1 item measure: How satisfied are you with your life as a whole?” on a 5-point Likert scale from 0 = ‘Not at all satisfied’ to 4 = ‘Very satisfied’ [5].

To assess mental well-being we used The World Health Organisation-Five Well-Being Index [1, 53]. The scale contains five items rated on a 6-point Likert scale from 0 = ‘At no time’ to 5 = ‘All the time’. One item was adapted to better suit the school-aged pupil population (the statement “I have felt active and vigorous” was changed to “I have felt active and energetic”). Total scores range from 0 to 100 (total raw score is multiplied by 4) and a higher score indicates better well-being.

Internal locus of control was assessed using a derived Locus of Control (LOC) scale adapted from the Department of Education report [17]. The scale contains three items rated on a 4-point Likert scale from 0 = ‘Strongly disagree’ to 3 = ‘Strongly agree’. Items included: ‘How much do you agree with this statement: ‘People like me don’t have much of a chance in life’, ‘How well you get on in this world is mostly a matter of luck’, ‘Even if I do well at school, I’ll have a hard time getting the right kind of job’. Total scores range from 0 and 9 and a higher score indicates a lower internal locus of control.

*Health behaviours.* Sleep was assessed by asking participants to indicate how many hours they typically slept per night with response options ‘less than 5 h’, ‘6–7’, ‘7–8’, ‘8–9’, ‘more than 9 h’.

Time spent using a screen (e.g., phone or computer) was assessed by asking participants to indicate how many hours they typically spent looking at screens per day with response options ‘1–4 h’, 5–7 h’, ‘more than 7 h’.

Advice seeking was assessed by the question: “Please finish this phrase “When I am stressed, anxious or in a low mood I tend to…” with response options 1 = ‘seek help and advice online’, 2 = ‘speak to friend(s)’, 3 = ‘speak to family member(s)’, 4 = ‘deal with it on my own’, 5 = ‘seek expert advice or counselling’).

### Ethics statement

The study was reviewed and approved through the formal Research Ethics procedure at the University of Sheffield (036380). Student participation was voluntary and anonymous, and all data was kept confidential.

### Data analysis

Table 2 summarises the data analysis methods used to assess the primary and secondary objectives. Descriptive statistics were used to present participant characteristics to show the demographic profile of the sample, and acceptability and feasibility outcomes. Participants who completed T1 and T2 were matched based on specified criteria (first two letters of first name, month of birth, age, gender, ethnic background, school) to assess the process of tracking participants longitudinally. Chi-square tests assessed differences in demographic characteristics for (a) responders, partial- and non-responders on mental health outcome measures; (b) matched participants, who completed T1 and T2, and non-matched participants who completed T1 only. Post hoc tests (z-tests of two proportions) were run for significant findings to explore the differences between categories. Bonferroni adjustments were made to account for multiple comparisons (a P-value of P < 0.0167 and P < 0.0125 was accepted for gender and ethnic background, respectively). Non-parametric Wilcoxon Signed-Rank tests comparing matched T1 and T2 data assessed differences in mental health outcome measures between T1 and T2. All statistical analyses used SPSS Version 26.0.

## Results

A total of 10,370 young people from 36 schools logged onto the OTL show via Slido at T1. Fifty-five responses were removed based on the exclusion criteria for age (> 18) resulting in 10,315 eligible participants who were included in the analysis for T1. A total of 3388 young people from 11 schools logged onto the OTL review show via Slido at T2. Nineteen responses were removed based on the exclusion criteria for age (> 18) resulting in 3369 eligible participants who were included in the analysis for T2. A total of 38 staff members completed the feedback survey and were included in the analysis at T1.

### Demographic characteristics

Baseline characteristics of the participants at baseline (T1) and follow-up (T2) are shown in Table 3. The sample was aged between 11 and 18 years at T1 (M = 13.56, SD = 1.36) and T2 (M = 13.53, SD = 1.23). A greater proportion of females than males took part in the intervention at both time points, and the proportion was most unequal at T2 (T1 F = 55%, T2 F = 62%). The majority were from White (60.7% at T1 versus 49.8% at T2) or Black (19% at T1 versus 24.3% at T2) ethnic backgrounds. Of the 36 schools that took part in T1, 17 schools were based in London and 19 schools were in Essex. Of the 11 schools that took part in T2, 8 schools were based in London and 3 schools were in Essex.

**Table 3:** Demographic information of the study sample at baseline and follow-up

Variable	Baseline (T1) Mean (SD) or* n* (%)	Total n	Follow-up (T2) Mean (SD) or* n* (%)	Total n
Schools enrolled		36		11
Age in years	13.56 (1.36)	7412	13.53 (1.23)	
Gender		7377		2619
Female	4050 (54.9%)		1623 (62.0%)	
Male	3015 (40.9%)		876 (33.4%)	
Prefer to self-identify	159 (2.2%)		74 (2.8%)	
Other	153 (2.1%)		46 (1.8%)	
Ethnicity		7404		2625
White	4497 (60.7%)		1306 (49.8%)	
Black/African/Caribbean/Black British	1405 (19.0%)		638 (24.3%)	
Asian/Asian British	586 (7.9%)		289 (11.0%)	
Mixed heritage	563 (7.6%)		244 (9.3%)	
Other	353 (4.8%)		148 (5.6%)	
Year group		7407		2631
Year 7 Age (in years)	1215 (16.4%) 11.76 (0.62)		397 (15.1%) 11.85 (0.52)	
Year 8 Age (in years)	1703 (23.0%) 12.70 (0.57)		685 (26.0%) 12.75 (0.54)	
Year 9 Age (in years)	2224 (30.0%) 13.69 (0.52)		860 (32.7%) 13.80 (0.49)	
Year 10 Age (in years)	1631 (22.0%) 14.68 (0.49)		604 (23.0%) 14.79 (0.47)	
Year 11 or above Age (in years)	634 (8.6%) 15.97 (1.05)		85 (3.2%) 16.19 (1.44)	

### Objective 1. Acceptability, engagement and demand of OTL intervention

Acceptability and engagement data for the OTL intervention collected from participants at T1 and T2 are shown in Table 4. Immediately after the OTL intervention at T1 the majority of participants reported that they found the OTL intervention engaging (88.5%) and felt more confident they had tools to help them better manage stress and anxiety (83.8%). Around three-quarters (76.1%) felt confident to offer advice to a friend who was feeling stressed. The majority of participants reported intent to try one or more of the mental health five-a-day techniques of creating headspace or doing something else (34%), a breathing technique (27.5%) or noticing what was going on for them at the time (18.3%). Almost half (48.3%) of participants reported they might use the Woebot app, whilst 38% would consider using the Kooth Online Counselling service. At T2 (4–6 weeks post-intervention), two-thirds of the sample (66.0%) reported that they viewed mental health in a more positive way and three-quarters (71.2%) reported that they now had a better understanding of what they need to do to maintain good mental health since they watched the show. Three quarters (75.6%) of young people also reported that they would like to see another OTL show. We assessed participant use of mental health services and tools at T2, where 10% of participants reported that since the show they had used the online counselling service, Kooth, one or more times and 34.7% used at least one of the OTL mental health 5-a-day tools.

**Table 4:** Acceptability and engagement of the OTL intervention post-intervention (n = 10,315) and at follow-up (N = 3369)

Time point	Outcome	Question	*Total n*	*n* (%) Agreeing	
1	Acceptability	Found the (OTL) show to be engaging	5576		
		Very – really felt involved Quite engaged – it was interesting A little – I was interested in some bits Not very – I didn’t feel engaged at all		958 2471 1506 641	(17.2%) (44.3%) (27.0%) (11.5%)
		Confidence participants had the tools to help manage stress and anxiety better	5510		
		Very confident Quite confident Would like more help		1288 3328 894	(23.4%) (60.4%) (16.2%)
		Mental health 5-a-day tool will be helpful	5540		
		Very helpful Helpful A little helpful Not helpful		756 1919 1986 879	(13.6%) (34.6%) (35.8%) (15.9%)
		Mental health 5-a-day might try 1 Noticing what is going on 2 Breathing technique 3 Grounding 4 Mindset (picking 3 focus words) 5 Headspace (doing something else)	5416	991 1487 463 632 1843	(18.3%) (27.5%) (8.5%) (11.7%) (34.0%)
		Likelihood of accessing Woebot app	5520		
		Will definitely use it Might consider it if I need it Probably won’t use it Won’t use it at all Will ask for help elsewhere		707 1961 1412 1186 254	(12.8%) (35.5%) (25.6%) (21.5%) (4.6%)
		Likelihood of using Kooth Online Counselling service^4^	5522		
		Will definitely use it Might consider it if I need it Probably won’t use it Won’t use it at all Will ask for help elsewhere		404 1715 1666 1440 297	(7.3%) (31.1%) (30.2%) (26.1%) (5.4%)
		Confident to offer advice to a friend who was feeling stressed to help them deal with it	5592		
		Confident I’d know what to do Still unsure		4182 1310	(76.1%) (23.9%)
2	Acceptability	“Since the On The Level show I view mental health in a more positive way”	2566		
		Strongly agree Kind of agree Kind of disagree Strongly disagree		278 1416 553 319	(10.8%) (55.2%) (21.6%) (12.4%)
		“Since the On The Level show I have a better understanding of what I need to do to maintain good mental health"	2565		
		Strongly agree Kind of agree Kind of disagree Strongly disagree		419 1407 493 246	(16.3%) (54.9%) (19.2%) (9.6%)
		Would like to see another On the Level show Definitely (yes) Maybe No	2407	911 909 587	(37.8%) (37.8%) (24.4%)
2	Engagement	Used a mental health 5-a-day tool 1 Noticing 2 Breathing technique 3 Grounding 4 Mindset 5 Headspace 6 None	2894	265 378 103 90 348 1829	(9.2%) (13.3%) (3.6%) (3.1%) (12.0%) (63.2%)
		Had a conversation about mental health^a^ 1 At school 2 With friend(s) 3 With family 4 With others	2662	223 1253 803 971	(8.4%) (47.1%) (30.2%) (36.5%)
		Used the Woebot app	2580		
		Signed up – using often Signed up – used a bit Signed up – did not use Did not sign up		60 168 242 2110	(2.3%) (6.5%) (9.4%) (81.8%)
		Signed up to Kooth Online Counselling	2564		
		Signed up during the OTL show Signed up after the OTL show Did not sign up Can’t remember		253 150 2127 34	(9.9%) (5.9%) (83.0%) (1.3%)
		Used Kooth Online Counselling	2591		
		Multiple times 2 or 3 times Once Not at all		39 54 177 2321	(1.5%) (2.1%) (6.8%) (89.6%)

^a^ Participants could select multiple response options for this item

#### Acceptability and ease of use data for staff members

Acceptability and ease of use data for staff members is presented in Table 6. The majority of staff members reported that they thought the OTL intervention was good or excellent (94.8%) and the approach taken to the mental health content covered was viewed positively (97.4%). The majority of staff members reported that accessing the link to the intervention was very easy (86.8%) and accessing the data collection tool, Slido, was easy for their students (92.1%). All staff members agreed that the information provided in OTL was important for young people in their school and that they would recommend the OTL intervention to other schools.

**Table 6:** Acceptability and ease of use of the OTL intervention for school staff members (n = 38)

Outcome	Question	*Total n*		*n* (%) Agreeing
Acceptability	What did you think of the show?	38		
	Excellent Good Ok Poor		21 15 2 0	(55.3%) (39.5%) (5.3%) (0.0%)
	How useful was the advice for young people?	37		
	Extremely useful Useful Quite useful Not very useful Would like more help		18 16 3 0 0	(48.6%) (43.2%) (8.1%) (0.0%) (0.0%)
	How well do you think the show approach the subjects covered?	38		
	The approach was ideal for students The approach was good It was okay Not the best approach The wrong approach		22 15 1 0 0	(57.9%) (39.5%) (2.6%) (0.0%) (0.0%)
	Would you recommend the show to other schools?	38		
	I would highly recommendation the show to other schools I would recommend the show to other schools I wouldn’t recommend the show to other schools		26 12 0	(68.4%) (31.6%) (0.0%)
	How important is the information in this show for young people in your school at this time?	38		
	Vital Important Not very important		26 12 0	(68.4%) (31.6%) (0.0%)
Ease of use	How easy did the students find accessing Slido?	38		
	Very easy Easy Needed more instruction Difficult		22 13 3 0	(57.9%) (34.2%) (7.9%) (0.0%)
	How good was the video quality?	38		
	Very high quality Somewhat high quality Good quality Somewhat low quality		21 13 3 1	(55.3%) (34.2%) (7.9%) (2.6%)

### Objective 2. Feasibility of implementing the intervention

#### Recruitment, reach and retention

**Fig. 2:**
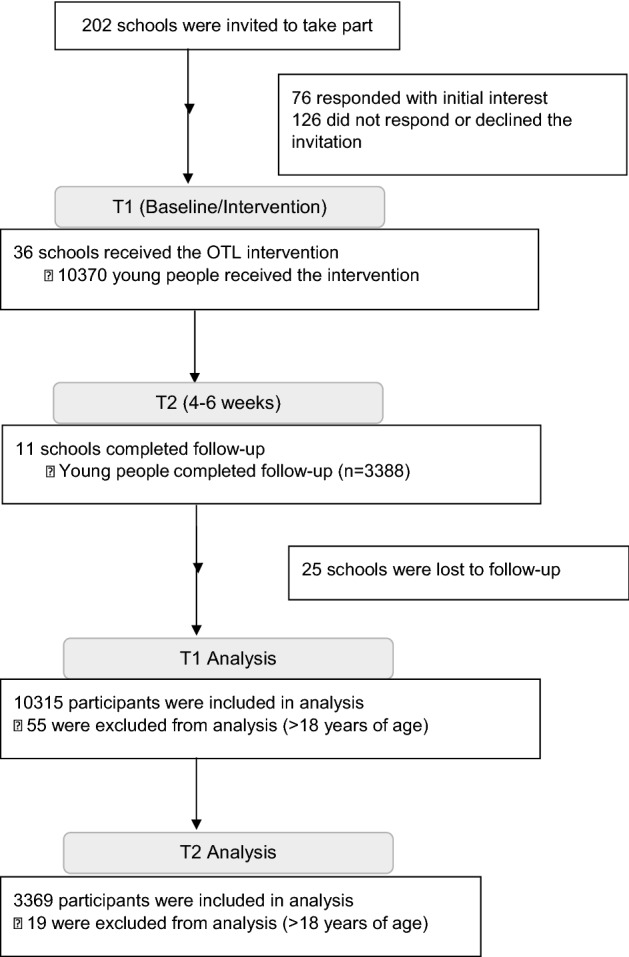
Study flow diagram – Recruitment, reach and retention. T1, Timepoint 1 (Baseline and Intervention); T2, Timepoint 2 (Follow-up 4–6 weeks post-intervention)

Recruitment, reach and retention of the OTL intervention at setting-level and individual-level are shown in the CONSORT-adapted flow diagram (Fig. 2). Seventy-six of 202 (37.6%) potentially eligible secondary schools responded with initial interest in the study. Thirty-six (of 76; 47.4%) schools enrolled in the OTL study completed the intervention (T1). Eleven of the 36 participating schools completed the follow-up session (T2) resulting in a retention rate of 30.6%. A total of 10,315 potentially eligible young people logged onto the OTL intervention at T1. Whilst 3,369 young people logged onto the OTL review show at T2, resulting in a follow-up rate of 32.7%.

#### Differential attrition

Some significant associations were observed between participants who were full responders compared to partial and non-responders, combined. Specifically, at T1 a greater proportion of full responders were female (*X*^*2*^ (2, N = 6659) = 8.17, p = 0.017) or from White ethnic backgrounds (*X*^*2*^ (3, N = 6362) = 18.65, p < 0.001). Whereas a higher proportion of partial- and non-responders included those who were male or from Black or Black British ethnic backgrounds. At T2, a greater proportion of full responders were from White ethnic backgrounds (*X*^*2*^ (3, N = 2460) = 16.84, p = 0.001). Whereas a greater proportion of partial or non-responders were from Black or Black British ethnic backgrounds. Differences between responders, and partial and non-responders are presented in Additional file 1: Table S4.

There were also significant associations between participants who were not matched (T1 only) compared to those who were matched (T1 and T2) on demographic measures collected at baseline (T1); specifically those who were matched were slightly younger in age (M = 13.42, SD = 1.11 versus M = 13.62, SD = 1.39, P < 0.01) and a greater proportion were female (*X*^*2*^ (2, N = 6659) = 84.76, p < 0.001) and from Asian or Asian British or Black or Black British backgrounds (*X*^*2*^ (3, N = 6362) = 58.92, p < 0.001). Whereas a greater proportion of those who were not matched identified as male or non-traditional and were from White ethnic backgrounds. Differences between matched and non-matched participants are presented in Additional file 1: Table S5.

### Objective 3. Feasibility of study methods

#### Data collection

**Fig. 3:**
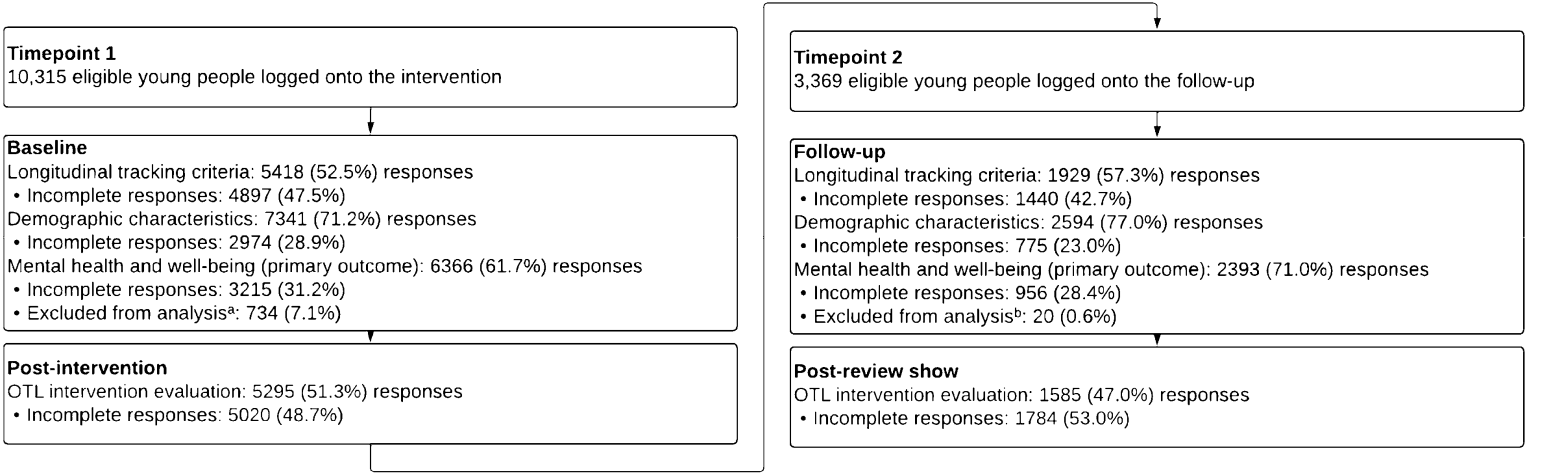
Proportion of participant responses and incomplete responses for survey measures collected at timepoint 1 baseline/intervention (n = 10,315) and timepoint 2 follow-up (N = 3369). ^a^Responses excluded from the analysis due to participants completing the items after the intervention. ^b^Responses excluded from the analysis due to participants completing the items after the ‘review show’ OTL, On the Level

The proportion of participant responses (and incomplete responses) for the baseline, post-intervention and follow-up survey measures are presented in Fig. 3. In a total sample size of 10,315 participants at T1, 28.9% of participant responses were incomplete (partial or non-responders) for demographic characteristics, 31.2% for mental health and well-being outcomes, and 48.7% for OTL evaluation items. In a total sample size of 3,369 participants at T2, 23% of participant responses were incomplete (partial or non-responders) for demographic characteristics, 28.4% for mental health outcomes, and 53% for OTL evaluation items.

Responses for mental health outcomes were removed at T1 (7.1%) for participants who completed the items after the intervention had taken place and at T2 (0.6%) for participants who completed the items after the review show had taken place. Whilst duplicate responses for mental health outcomes were observed at T1 (1.5%) and T2 (0.1%).

#### Longitudinal tracking

A total of 759 (22.5%) participants (out of 3,369 young people who logged onto the follow-up session at T2) were matched for responses at T1 and T2. Overall, 77.5% participants at T2 were not matched with T1 responses due to missing data on one or more of the matching criteria items.

### Objective 4. Need for mental health intervention and feasibility of outcome measures

The need for the OTL intervention was supported by the large percentage of students reporting poor mental health and poor health behaviour at T1 (see Table 5; data was collected at baseline before the start of the OTL show). Specifically, almost 40% experienced anxiety (either all the time, most of the time, or more than half the time) and 20% experienced depression (either all the time, most of the time, or more than half the time). Using a cut-off of 9 for the perceived scale for our sample revealed that 31.5% experienced high levels of perceived stress (scores 7–9), and 50% experienced moderate perceived stress (scores 5–8) (quartile scores for this scale for our sample—25th: 5; 50th: 7; 75th: 9). In line with 37% reported struggling or experiencing critical levels of stress using our stress dial measure. In our sample there were also very high numbers of young people who experienced very low locus of control, for example almost 70% reporting that ‘People like me don’t have much of a chance in life’. In addition, large number of young people surveyed reported poor health-behaviours, with a high number reporting low levels of sleep (45% less than 7 h sleep) and very high levels of screen time use (33.2% more than 7 h/day).

**Table 5:** Mental health, well-being and health-behaviour measures for all participants at baseline T1 (n = 10,315)

Measure	Range	Baseline Median (IQR) or n (%)	N
Anxiety	0–5	1.00 (2.0)	6672
All the time		464 (7.0%)	
Most of the time		1052 (15.8%)	
More than half the time		955 (14.3%)	
Less than half the time		830 (12.4%)	
Some of the time		2115 (31.7%)	
At no time		1256 (18.8%)	
Depression	0–5	1.00 (2.0)	6662
All the time		259 (3.9%)	
Most of the time		521 (7.8%)	
More than half the time		654 (9.8%)	
Less than half the time		673 (10.1%)	
Some of the time		1624 (24.4%)	
At no time		2931 (44.0%)	
Life satisfaction	0–4	3.00 (1.0)	6670
Very satisfied		1028 (15.4%)	
Satisfied		2875 (43.1%)	
Neither satisfied nor unsatisfied		2004 (30.0%)	
Not very satisfied at all		549 (8.2%)	
Not at all satisfied		214 (3.2%)	
Well-being	0–100	52.00 (40.0)	6607
Perceived stress	0–16	7.00 (4.0)	6554
Stress dial	1–4	2.00 (1.0)	7763
Green (healthy)		1875 (24.2%)	
Yellow (coping)		3165 (40.8%)	
Orange (struggling)		1903 (24.5%)	
Red (critical)		820 (10.6%)	
Locus of control	0–9	4.00 (3.0)	6562
**‘**People like me don’t have much of a chance in life’			
Strongly disagree Kind of disagree Kind of agree Strongly agree		2453 (37.4%) 2090 (31.9%) 1664 (25.4%) 355 (5.4%)	
‘How well you get on in this world is mostly a matter of luck’			
Strongly disagree Kind of disagree Kind of agree Strongly agree		1223 (18.6%) 2357 (35.9%) 2493 (38.0%) 489 (7.5%)	
‘Even if I do well at school, I’ll have a hard time getting the right kind of job’			
Strongly disagree Kind of disagree Kind of agree Strongly agree		797 (12.1%) 1899 (28.9%) 2937 (44.8%) 929 (14.2%)	
Sleep *(in hours)*	1–5	3.00 (1.0)	6650
Less than 5		753 (11.3%)	
6–7 h		2264 (34.0%)	
7–8 h		2105 (31.7%)	
8–9 h		1197 (18.0%)	
More than 9		331 (5.0%)	
Screen time *(in hours)*	1–3	2.00 (2.0)	6662
1–4		1763 (26.5%)	
5–7		2687 (40.3%)	
More than 7		2212 (33.2%)	

Comparisons were made on mental health outcome measures for matched participants between T1 and T2 (shown in Additional file 1: Table S7). There were some significant differences found between T1 and T2; specifically, Wilcoxon signed-rank tests determined there was a statistically significant median decrease is stress levels as assessed by the stress dial (z = -5.19, P < 0.001) between T1 and T2. There was also a slight increase in anxiety (z = 2.03, P = 0.043) and depression (z = 3.26, P = 0.001) scores between T1 and T2; and a slight decrease in life satisfaction (z = -2.26, P = 0.024) scores. There were no statistically significant differences for well-being, perceived stress and locus of control from T1 to T2.

## Discussion

### Acceptability, engagement, and demand

The results suggest high acceptability of the OTL intervention among young people and staff members, with a large proportion of participants finding the intervention engaging and reporting they would like to see another OTL show. Importantly, the proportion of young people who engaged with NHS commissioned digital health services, such as the Kooth online counselling service, in this study was found to be at similar levels to the proportion of young people presenting mental health disorders in previous research [23], adding further support for the need of the delivery of the OTL mental health intervention in secondary schools. Our findings show that OTL can effectively help schools provide mental health education and support for their students and help address schools statutory Safeguarding and PSHE obligations (Keeping Children Safe in Education (KCSIE, 2018) to identify concerns early and provide help for youth in this area (KCSIE1.4, 1.6). Moreover, OTL is in accordance with KCSIE framework that schools work with other services to promote the welfare of youth and protect them from harm (KCSIE 1.81) and addresses key elements of the Government’s COVID-19 Mental Health and Wellbeing recovery action plan (March 2021).

Our baseline mental health and well-being survey (T1) revealed high numbers of young people experiencing low levels of well-being, especially in their reported mental health levels relating to stress and anxiety. Using the stress dial over a third of young people reported they were struggling or experiencing critical levels of stress, which notably we found reduced to a quarter of the sample 4–6 weeks after the show (T2). The majority of young people also reported not getting enough sleep and very high levels of screen time, both of which have been shown to have an adverse impact on well-being [41, 49]. All the above was also accompanied with very low levels of internal locus of control. For example, a third of young people reported that “*people like me don’t have much of a chance in life*”, and three quarters of young people reported that “*even if I did well at school I would have a hard time getting the right kind of job*”. This is of particular concern, as there is strong evidence that low locus of control is associated with poor mental health [11, 40, 46]. Together, these findings illustrate the critical need for OTL mental health intervention to support young people in secondary schools.

Feedback from the staff members, who facilitated the delivery of OTL in class, also supported the high acceptability and importance of the OTL intervention for young people. Staff feedback confirmed the OTL intervention could be effectively delivered in classrooms as planned with the majority of staff members finding it easy to access the link to the intervention and the data collection tool, Slido. This is a positive finding with previous issues documented around intervention implementation related to teacher burden and/or lack of mental health training [27]. This will be important for engaging schools and their staff members in the provision of the OTL intervention to young people in future research as the intervention does not require staff to have specific expertise or training in mental health.

### Feasibility of implementing the intervention

The feasibility of implementing the OTL intervention at a setting-level indicated almost half of eligible schools completed the intervention at T1 which was slightly lower than other universal approaches with recruitment rates of between 67 and 88% [43]. Further, a large proportion of secondary schools (69.4%) did not return to follow-up resulting in high attrition rates between T1 and T2. This finding is consistent with previous studies reporting high levels of attrition in universal school-based mental health interventions [19, 24] with some studies experiencing challenges with investment from schools in terms of participation and data collection at follow-up [19, 27, 43]. This could have, in part, been affected by the increased demands faced by schools during the pandemic which may have affected school capacity and engagement with the intervention. Both the recruitment and retention of schools between intervention and follow-up needs to be considered for a future randomised controlled trial (RCT) of OTL to explore ways to engage schools and the relevant staff members from the initial contact through to follow-up. For example, attending relevant school meeting networks or building connections with mental health and well-being personnel within the targeted schools.

The results show that it is possible to reach a large sample of young people from different academic year groups and to roll out the OTL intervention programme to secondary schools across the UK based on the large sample size recruited in this study. Thus, our study indicated that providing a live on-line 50-min interactive (by use of engaging with the digital data collection tool, Slido) broadcast of a previously filmed digital mental health intervention content during class is feasible for reaching the target population.

Differential attrition was found for participants who responded fully to the mental health outcomes compared to those who responded partially or not at all. Differences in demographic characteristics for the completion of key outcome measures need to be considered more fully in a future RCT of the OTL intervention to increase the likelihood of recruiting a representative sample from the participating schools. Future studies of the OTL intervention should consider oversampling for males and those from Black or Black British ethnic backgrounds. Similarly, differences were observed for participants who were not matched and those who were matched at T1 and T2, with those who are older, identify as male or non-traditional and those from White ethnic backgrounds less likely to be matched across timepoints. However, the differences observed between matched and non-matched participants need to be considered with caution as there was a much higher proportion of participants who attended follow-up (T2) than were able to be matched due to missing data for the specified matching criteria. Further, we were unable to determine whether those less likely to be matched at T1 and T2 was due to missing data for the matching criteria or whether certain groups were less likely to take part in the follow-up session.

### Feasibility of study methods

In line with this, only a small proportion of participants were successfully matched between T1 and T2, suggesting the criteria employed to longitudinally track and match participants across timepoints should be adapted to better suit this population. We used the first two letters of the first name to match participant data at T1 and T2. However, large quantities of missing data and possible typing errors or misinterpretation for this item meant it was difficult to match participants for comparison on mental health outcomes at T1 and T2. This would be problematic in a larger-scale evaluation assessing the effectiveness of the intervention, hence future work should incorporate better unique identifiers, without compromising the anonymity of the young people taking part.

On inspection of the missing data observed in the current study, completion rates for the study measures varied with an increasing proportion of missing items observed as the intervention proceeded, with the highest proportion of missing data observed for the post-intervention and follow-up evaluation items. This finding is consistent with considerations around conducting research with children and young people and specifically related to the length and complexity of surveys disseminated to different ages groups [39]. Missing data for the mental health and well-being outcome measures needs to be considered further to ensure primary outcome data can be successfully collected in a RCT of OTL if we are to assess the efficacy and effectiveness of the intervention. Collecting study data at the beginning of the session as we did in this study might reduce the likelihood of large amounts of missing data for primary outcome data and help to maximise data completeness whilst participants are more engaged. The majority of staff members adhered to the data collection procedure ensuring that the relevant surveys were provided and completed by students at the appropriate timepoints during the study.

### Study strengths and limitations

This feasibility study had several strengths. We recruited a large sample of young people between the ages 11–18 years from secondary schools across London and Essex in the UK. The study was carried out in a real-life setting using existing resources and availability within the secondary school system. We used a broad range of outcome measures to explore different aspects of mental health and well-being. We used an interactive data collection tool to promote interaction and engagement during the intervention and to respond to the need to move away from traditional pen-to-paper methods of data collection with young people (Garrido et al. 2019). Finally, due to the pre-filmed nature of the OTL mental health intervention, all students received the same mental health intervention for the specified 50-min duration presented by the same young presenters.

The study has various limitations. The intervention was rolled out universally to young people at secondary school in the UK and we were unable to include a comparison or control group in this study. Assessing the limited efficacy of the intervention in this feasibility study cannot therefore be fully examined. Future research should include a comparison group matched on key demographic measures and randomly allocate participants to the intervention or control group. Further, we used 1-item questions to measure anxiety, depression, and life satisfaction intended to detect changes in outcomes between T1 and T2 which may be insufficient. We recommend that for more robust evaluation of the intervention, future studies should use validated outcome measures with clinical cut-offs so that the clinical significance of any changes can be assessed. For example, the Patient Health Questionnaire-9 (PHQ-9; Kroenke, Spitzer, & Williams, 2001) for depression, General Anxiety Disorder scale (GAD-7; Spitzer, Kroenke, Williams, & Löwe, 2006) for anxiety, and the Warwick-Edinburgh Mental Well-being scale (WEMWBS; Tennant et al., 2017) for well-being are extensively used in mental health research and may therefore be more appropriate. The length and complexity of surveys used with children and young people should, however, be considered. Previous research suggests that lengthy surveys and complex questions can impact response rates and attrition among children and young people (Borgers, Leeuw & Hox, 2000; Borgers, Sikkel & Hox, 2004).

In addition, the findings need to be considered in the light of the high attrition rate between T1 and T2. Only 30% of the schools where students received the OTL intervention at T1, were able to take part in our follow up review show at T2, where we collected outcome data. The high attrition resulting from this, may have resulted in a selection bias. Schools that did not take part in T2, may have been schools that were under additional pressure, as were students in these schools, which could have meant that the impact of OTL on the missing demographic may have been significantly different. As we cannot remove potential effects of section bias from this study, this limitation will be addressed in future a RCT study of OTL.

### Future research

Overall, there was good evidence to suggest mental health interventions are needed considering the high levels of possible anxiety, depression and low internal locus of control reported among young people in this study. The OTL intervention, using a cognitive behavioural framework, worked on trying to break down the stigma around mental health and well-being and other potential barriers which may prevent young people from accessing mental health support and engaging in mental health-related conversations [20, 32]. Universally delivered mental health interventions, such as OTL, are therefore important for providing mental health education, preventive tools and access to mental health support [20]. Further investigation on the OTL intervention in a RCT study would be key and would help to determine the efficacy and potential effectiveness of an interactive mental health intervention delivered to young people in secondary schools.

The present study included young people aged between 11 and 18 years, however young people in earlier years may have different needs, preferences and experiences compared with those in the latter years [36], this needs further investigation and co-creation with adolescents of different ages and/or developmental timepoints. In the future, it will also be important to investigate the reasons why some young people may not take part or are not fully engaged in this type of intervention [9]. For instance, qualitative interview techniques or focus groups would further inform our understanding of the acceptability of the OTL intervention along with the study methods employed in this study. Qualitative information would help inform future iterations of the OTL intervention including the topic areas viewed as most important to young people. It is also important to note that there was also COVID-19-related content within the present intervention, however there will be less focus on pandemic related content in future iterations of OTL. A strength of the OTL intervention is that due to its dynamic method of delivery along with co-creation with young people, the content can be modified to respond to the emerging needs of young people. Co-development of mental health programmes is valuable to ensure that interventions are relevant, informed by the target users, and are effective [13]. Hence, the OTL intervention is very adaptable and modifiable to different needs, preferences, and experiences and would benefit from additional evaluation. As such, following the provision of the initial OTL mental health intervention for this study, updated versions of OTL have been developed and delivered. For instance, it was found that the Woebot application may not be appropriate for this population [47] and is therefore no longer included in future iterations of the OTL mental health intervention.

## Conclusion

This study provides strong feasibility, acceptability and need of OTL mental health intervention, and supports the rolling out of this intervention to young people as part of a preventive mental health strategy delivered in secondary schools. Some adaptations to the study methods and implementation of the intervention in schools were identified which will help inform future research on the OTL intervention, and based on the findings of this feasibility study, a larger RCT study is needed to further determine the effectiveness of the intervention.

## Supplementary Information

**Additional file 1. Supplementary Information**

## Data Availability

Dataset and materials can be accessed on the OSF (URL, to come).
