# Supplementary material for: Feasibility of a secondary school-based mental health intervention: Reprezents’ On The Level
Source: Child Adolesc Psychiatry Ment Health. 2022 Dec 9;16:98. doi: 10.1186/s13034-022-00534-2 (PMC9735021; doi:10.1186/s13034-022-00534-2)
Supplement: Supplementary file 1 — Additional file 1. Supplementary Information [file 13034_2022_534_MOESM1_ESM.docx]

**Supplementary Information**

**Supplementary Table 1.** Survey measures collected during the On the Level intervention at Timepoint 1

| **Question** | **Response options** |
| --- | --- |
| ‘I have my own critical voices’ | -Yes  -No  -Maybe |
| One negative thing your critical voices attack | -The way you look  -Your personality  -Whether you're likeable or loveable  -Trust in others  -Your ability to succeed at anything  -All of you |
| ‘When I emotionally overload, I tend to...’ | -Implode  -Explode |
| Which level do you feel you are on at the moment in that stress dial? | -Green – Healthy  -Yellow – Coping  -Orange – Struggling  -Red – Critical |
| What level were you on in the last lockdown in that stress dial? | -Green – Healthy  -Yellow – Coping  -Orange – Struggling  -Red – Critical |
| What are the mental health 5-a-day? | -Make your body heavy  -Sit and chill  -Headspace  -Watch TV  -Notice  -Breathe out slower  -Growth mindset-3 words  -Binge eat |

**Supplementary Table 2.** Acceptability of the On the Level intervention measures collected at post-intervention (timepoint 1) and follow-up (timepoint 2)

| **Timepoint** | **Question** | **Response options** |
| --- | --- | --- |
| 1 | How engaging did you find the show? | -Not very – I didn’t feel engaged at all  -A little – I was interested in some bits  -Quite engaged - it was interesting  -Very - really felt involved |
|  | How confident do you feel you have the tools to help you manage stress and anxiety better? | -Would like more help  -Quite confident  -Very confident |
|  | How helpful do you think the mental health 5 a day will be for you? | 1 - Not helpful  2 - A little helpful  3 - Helpful  4 - Very helpful |
|  | Which of the mental health 5-a-day do you think you might try? | -Noticing what's going on with me  -Slowing down my breathing out  -Dropping my weight into the ground or the chair  -Mindset - pick three focus words  -Headspace - do something else for a bit |
|  | How likely are you to access the Woebot app | -Will definitely use it  -Might consider it if I need it  -Probably won't use it  -Won't use it at all  -Will ask for help elsewhere |
|  | How likely are you to use the Kooth Online Counselling service? | -I will definitely use it  -Might consider it if needed  -Probably won't use it  -Won't use it at all  -Will ask for help elsewhere |
|  | If a friend was feeling stressed, how confident would you feel about offering them advice to help them deal with it now you've seen this On The Level mental health show by Reprezent? | -Confident I'd know what to do  -Still Unsure |
| 2 | Since the On the Level show, I view mental health in a more positive way | 1 – Strongly disagree  2 – Kind of disagree  3 – Kind of agree  4 – Strongly agree |
|  | Since the On The Level show, I have a better understanding of what I need to do to maintain good mental health | 1 – Strongly disagree  2 – Kind of disagree  3 – Kind of agree  4 – Strongly agree |
|  | Since the last show have you used any of these tools? | -Headspace (go out and do something different for a bit)  -3 positive words  -Dropping your body weight into the ground/ a chair  -Breathing out slower  -Noticing what's going on with me (being aware of the signs)  -None of these |
|  | Since the last show I have had conversations about mental health with ^a^ … | -others (yes/ no)  -family (yes/no)  -friends (yes/ no)  -in school (yes/no) |
|  | Have you used Woebot? | -I didn’t sign up to Woebot  -I signed up to Woebot but didn't use it  -I signed up to Woebot and have used a bit  -I signed up to Woebot and have been using it often |
|  | Did you sign up to Kooth during or after the last On The Level Show? | -I didn't sign up to Kooth  -I signed up after the show  -I signed up during the On the Level show  -Can't remember |
|  | Have you used Kooth since? | -Not at all  -Once  -Two or Three Times  -Multiple times |
|  | Would you like to see another On The Level show? | -No  -Maybe  -Definitely Yes |

^a^ Participants could select multiple response options for this item

**Supplementary Table 3.** Measures for acceptability, ease of use and importance of the OTL intervention for school staff members (timepoint 1)

| **Question** | **Response options** |
| --- | --- |
| What did you think of the show? | -Excellent  -Good  -Ok  -Poor |
| How useful was the advice for young people? | -Extremely useful  -Useful  -Quite useful  -Not very useful |
| How well do you think the show approach the subjects covered? | -The approach was ideal for students  -The approach was good  -It was okay  -Not the best approach  -The wrong approach |
| How easy was the show private YouTube link to access? | -Very easy  -Needs more instruction  -Very difficult |
| How easy did the students find accessing Slido? | -Very easy  -Easy  -Needed more instruction  -Difficult |
| How good was the video quality? | -Very high quality  -Somewhat high quality  -Good quality  -Somewhat low quality |
| How important is the information in this show for young people in your school at this time? | -Vital  -Important  -Not very important |
| Would you recommend the show to other schools? | -I would highly recommendation the show to other schools  -I would recommend the show to other schools  -I wouldn’t recommend the show to other schools |

**Supplementary Table 4.** Differences in demographic characteristics between responders and partial or non-responders on mental health outcomes at baseline (n = 10315) and follow-up (N = 3369)

| Demographic characteristic | Baseline: T1 | | | |  | Follow-up: T2 | | | |  |
| --- | --- | --- | --- | --- | --- | --- | --- | --- | --- | --- |
|  | **Responders**  **n (%)** | | **Partial & non-responders**  **n (%)** | | **p-value** | **Responders**  **n (%)** | | **Partial & non-responders**  **n (%)** | | **p-value** |
| Gender |  |  |  |  |  |  |  |  |  |  |
| Female | 3524 | (95.9%) | 152 | (4.1%) | **0.005** | 1478 | (91.5%) | 137 | (8.5%) | 0.966 |
| Male | 2566 | (94.4%) | 152 | (5.6%) | **0.013** | 788 | (91.1%) | 77 | (8.9%) | 0.572 |
| Non-traditional | 249 | (94.0%) | 16 | (6.0%) | 0.0339 | 113 | (95.0%) | 6 | (5.0%) | 0.170 |
| Ethnicity |  |  |  |  |  |  |  |  |  |  |
| Asian/Asian British | 527 | (96.3%) | 20 | (3.7%) | 0.204 | 260 | (90.0%) | 29 | (10.0%) | 0.368 |
| Black/Black British | 1209 | (93.0%) | 91 | (7.0%) | **< 0.001** | 565 | (88.6%) | 73 | (11.4%) | **< 0.001** |
| White | 3856 | (95.8%) | 169 | (4.2%) | **0.006** | 1214 | (93.0%) | 92 | (7.0%) | **< 0.001** |
| Mixed Heritage | 467 | (95.3 %) | 23 | (4.7%) | 0.941 | 223 | (91.4%) | 21 | (8.6%) | 0.728 |

Significant results in Bold type

**Supplementary Table 5.** Differences in demographic characteristics between matched (timepoint 1 and 2) and non-matched (timepoint 1 only) participants at baseline

| Demographic characteristic | Non-matched  n (%) | | Matched  n (%) | | p-value |
| --- | --- | --- | --- | --- | --- |
| Gender |  |  |  |  |  |
| Female | 3153 | (85.8%) | 523 | (14.2%) | **< 0.001** |
| Male | 2517 | (92.6%) | 201 | (7.4%) | **< 0.001** |
| Non-traditional | 252 | (95.1%) | 13 | (4.9%) | **0.001** |
| Ethnicity |  |  |  |  |  |
| Asian/Asian British | 447 | (81.7%) | 100 | (18.3%) | **< 0.001** |
| Black/Black British | 1110 | (85.4%) | 190 | (14.6%) | **< 0.001** |
| White | 3649 | (90.7%) | 376 | (9.3%) | **< 0.001** |
| Mixed Heritage | 445 | (90.8%) | 45 | (9.2%) | 0.145 |

Significant results in Bold type

**Supplementary Table 6.** Mental health, well-being and health-related behaviour measures at baseline (n = 10315) and follow-up (N = 3369)

| Measure | Range | Baseline Median (IQR) or n (%) | N | Follow-up Median (IQR) or n (%) | N |
| --- | --- | --- | --- | --- | --- |
| Anxiety | 0-5 | 1.00 (2.0) | 6672 | 2.00 (3.0) | 2579 |
| All the time |  | 464 (7.0%) |  | 206 (8.0%) |  |
| Most of the time |  | 1052 (15.8%) |  | 448 (17.4%) |  |
| More than half the time |  | 955 (14.3%) |  | 460 (17.8%) |  |
| Less than half the time |  | 830 (12.4%) |  | 417 (16.2% |  |
| Some of the time |  | 2115 (31.7%) |  | 681 (26.4%) |  |
| At no time |  | 1256 (18.8%) |  | 367 (10.9%) |  |
| Depression | 0-5 | 1.00 (2.0) | 6662 | 1.00 (3.0) | 2575 |
| All the time |  | 259 (3.9%) |  | 127 (4.9%) |  |
| Most of the time |  | 521 (7.8%) |  | 278 (10.8%) |  |
| More than half the time |  | 654 (9.8%) |  | 306 (11.9%) |  |
| Less than half the time |  | 673 (10.1%) |  | 366 (14.2%) |  |
| Some of the time |  | 1624 (24.4%) |  | 531 (20.6%) |  |
| At no time |  | 2931 (44.0%) |  | 967 (37.6%) |  |
| Life satisfaction | 0-4 | 3.00 (1.0) | 6670 | 3.00 (1.0) | 2587 |
| Very satisfied |  | 1028 (15.4%) |  | 380 (14.7%) |  |
| Satisfied |  | 2875 (43.1%) |  | 1021 (39.5%) |  |
| Neither satisfied nor unsatisfied |  | 2004 (30.0%) |  | 804 (31.1%) |  |
| Not very satisfied at all |  | 549 (8.2%) |  | 258 (10.0%) |  |
| Not at all satisfied |  | 214 (3.2%) |  | 124 (4.8%) |  |
| Well-being | 0-100 | 52.00 (40.0) | 6607 | 52.00 (36.0) | 2535 |
| Perceived stress | 0-16 | 7.00 (4.0) | 6554 | 8.00 (3.0) | 2506 |
| Stress dial | 1-4 | 2.00 (1.0) | 7763 | 2.00 (1.0) | 2880 |
| Green (healthy) |  | 1875 (24.2%) |  | 702 (24.4%) |  |
| Yellow (coping) |  | 3165 (40.8%) |  | 1321 (45.9%) |  |
| Orange (struggling) |  | 1903 (24.5%) |  | 594 (20.6%) |  |
| Red (critical) |  | 820 (10.6%) |  | 263 (9.1%) |  |
| Locus of control | 0-9 | 4.00 (3.0) | 6562 | 4.00 (4.0) | 2482 |
| Sleep *(in hours)* | 1-5 | 3.00 (1.0) | 6650 | 3.00 (1.0) | 2530 |
| Less than 5 |  | 753 (11.3%) |  | 345 (13.6%) |  |
| 6-7 hours |  | 2264 (34.0%) |  | 884 (34.9%) |  |
| 7-8 hours |  | 2105 (31.7%) |  | 756 (29.9%) |  |
| 8-9 hours |  | 1197 (18.0%) |  | 428 (16.9%) |  |
| More than 9 |  | 331 (5.0%) |  | 117 (4.6%) |  |
| Screen time *(in hours)* | 1-3 | 2.00 (2.0) | 6662 | 2.00 (2.0) | 2522 |
| 1-4 |  | 1763 (26.5%) |  | 696 (27.6%) |  |
| 5-7 |  | 2687 (40.3%) |  | 1029 (40.8%) |  |
| More than 7 |  | 2212 (33.2%) |  | 797 (31.6%) |  |

**Supplementary Table 7.** Comparisons of changes in mental health outcomes between baseline and follow-up for matched participants (N=759)

|  | |  | |  | |  | |  |  |  |
| --- | --- | --- | --- | --- | --- | --- | --- | --- | --- | --- |
| Measure | | **Range** | | **Baseline (T1)**  **Median (IQR range)**  **or *n(%)*** | **Follow-up (T2)**  **Median (IQR range)**  **or *n(%)*** | | **n** | | **Z** | **p-value** |
| Anxiety | | 0-5 | | 2.00 (2.0) | 2.00 (2.0) | | 724 | | **2.025** | **0.043** |
| All the time | |  | | 40 (5.5%) | 43 (5.9%) | |  | |  |  |
| Most of the time | |  | | 125 (17.3%) | 121 (16.7%) | |  | |  |  |
| More than half the time | |  | | 123 (17.0%) | 131 (18.1%) | |  | |  |  |
| Less than half the time | |  | | 79 (10.9%) | 125 (17.3%) | |  | |  |  |
| Some of the time | |  | | 230 (31.8%) | 200 (27.6%) | |  | |  |  |
| At no time | |  | | 127 (17.5%) | 104 (14.4%) | |  | |  |  |
| Depression | | 0-5 | | 1.00 (2.0) | 1.00 (2.0) | | 721 | | **3.263** | **0.001** |
| All the time | |  | | 14 (1.9%) | 25 (3.5%) | |  | |  |  |
| Most of the time | |  | | 68 (9.4%) | 62 (8.6%) | |  | |  |  |
| More than half the time | |  | | 61 (8.5%) | 79 (11.0%) | |  | |  |  |
| Less than half the time | |  | | 95 (13.2%) | 123 (17.1%) | |  | |  |  |
| Some of the time | |  | | 182 (25.2%) | 145 (20.1%) | |  | |  |  |
| At no time | |  | | 301 (41.7%) | 287 (39.8%) | |  | |  |  |
| Life satisfaction | | 0-4 | | 3.00 (1.0) | 3.00 (1.0) | | 731 | | **-2.260** | **0.024** |
| Very satisfied | |  | | 106 (14.5%) | 107 (14.6%) | |  | |  |  |
| Satisfied | |  | | 334 (45.7%) | 312 (42.7%) | |  | |  |  |
| Neither satisfied nor unsatisfied | |  | | 211 (28.9%) | 212 (29.0%) | |  | |  |  |
| Not very satisfied at all | |  | | 62 (8.5%) | 71 (9.7%) | |  | |  |  |
| Not at all satisfied | |  | | 18 (2.5%) | 29 (4.0%) | |  | |  |  |
| Well-being | | 0-100 | | 52.00 (40.0) | 52.00 (40.0) | | 713 | | 0.979 | 0.328 |
| Perceived stress | | 0-16 | | 7.00 (4.0) | 7.00 (4.0) | | 708 | | 0.531 | 0.595 |
| Stress dial | | 1-4 | | 2.00 (1.0) | 2.00 (1.0) | | 579 | | **-5.194** | **< 0.001** |
| Green (healthy) | |  | | 118 (20.4%) | 145 (25.0%) | |  | |  |  |
| Yellow (coping) | |  | | 250 (43.2%) | 291 (50.3%) | |  | |  |  |
| Orange (struggling) | |  | | 158 (27.3%) | 105 (18.1%) | |  | |  |  |
| Red (critical) | |  | | 53 (9.2%) | 38 (6.6%) | |  | |  |  |
| Locus of control | | 0-9 | | 4.00 (3.0) | 4.00 (3.0) | | 704 | | -0.239 | 0.811 |
| ‘People like me don’t have much of a chance in life’ |  | | | |  | |  | |  |  |
| Strongly disagree | |  | | 260 (36.9%) | 280 (39.8%) | |  | |  |  |
| Kind of disagree | |  | | 235 (33.4%) | 206 (29.3%) | |  | |  |  |
| Kind of agree | |  | | 164 (23.3%) | 176 (25.0%) | |  | |  |  |
| Strongly agree | |  | | 45 (6.4%) | 42 (6.0%) | |  | |  |  |
| ‘How well you get on in this world is mostly a matter of luck’ |  | | | |  | |  | |  |  |
| Strongly disagree | |  | | 146 (20.7%) | 146 (20.7%) | |  | |  |  |
| Kind of disagree | |  | | 261 (37.1%) | 244 (34.7%) | |  | |  |  |
| Kind of agree | |  | | 261 (37.1%) | 266 (37.8%) | |  | |  |  |
| Strongly agree | |  | | 36 (5.1%) | 48 (6.8%) | |  | |  |  |
| ‘Even if I do well at school, I’ll have a hard time getting the right kind of job’ | | |  | | | |  | |  |  |
| Strongly disagree | |  | | 87 (12.4%) | 97 (13.8%) | |  | |  |  |
| Kind of disagree | |  | | 216 (30.7%) | 194 (27.6%) | |  | |  |  |
| Kind of agree | |  | | 289 (41.1%) | 326 (46.3%) | |  | |  |  |
| Strongly agree | |  | | 112 (15.9%) | 87 (12.4%) | |  | |  |  |
| Sleep *(in hours)* | | 1-5 | | 3.00 (1.0) | 3.00 (1.0) | | 716 | | -1.084 | 0.278 |
| Less than 5 | |  | | 81 (11.3%) | 84 (11.7%) | |  | |  |  |
| 6-7 hours | |  | | 248 (34.6%) | 252 (35.2%) | |  | |  |  |
| 7-8 hours | |  | | 232 (32.4%) | 236 (33.0%) | |  | |  |  |
| 8-9 hours | |  | | 123 (17.2%) | 118 (16.5%) | |  | |  |  |
| More than 9 | |  | | 32 (4.5%) | 26 (3.6%) | |  | |  |  |
| Screen time *(in hours)* | | 1-3 | | 2.00 (2.0) | 2.00 (2.0) | | 717 | | -1.514 | 0.130 |
| 1-4 | |  | | 185 (25.8%) | 184 (25.7%) | |  | |  |  |
| 5-7 | |  | | 284 (39.6%) | 314 (43.8%) | |  | |  |  |
| More than 7 | |  | | 248 (34.6%) | 219 (30.5%) | |  | |  |  |

Significant findings are in bold type. Z is the Standardized Test Statistic.
